# Pharmacodynamic and pharmacokinetic neoadjuvant study of hedgehog pathway inhibitor Sonidegib (LDE-225) in men with high-risk localized prostate cancer undergoing prostatectomy

**DOI:** 10.18632/oncotarget.22115

**Published:** 2017-10-26

**Authors:** Ashley E. Ross, Robert M. Hughes, Stephanie Glavaris, Kamyar Ghabili, Ping He, Nicole M. Anders, Rana Harb, Jeffrey J. Tosoian, Luigi Marchionni, Edward M. Schaeffer, Alan W. Partin, Mohamad E. Allaf, Trinity J. Bivalacqua, Carolyn Chapman, Tanya O’Neal, Angelo M. DeMarzo, Paula J. Hurley, Michelle A. Rudek, Emmanuel S. Antonarakis

**Affiliations:** ^1^ James Buchanan Brady Urological Institute, Department of Urology, Johns Hopkins School of Medicine, Baltimore, MD, USA; ^2^ Department of Oncology, Johns Hopkins School of Medicine, Baltimore, MD, USA; ^3^ Sidney Kimmel Cancer Center, Johns Hopkins School of Medicine, Baltimore, MD, USA; ^4^ Analytical Pharmacology Core Laboratory, Sidney Kimmel Comprehensive Cancer Center at Johns Hopkins, Baltimore, MD, USA; ^5^ Department of Pathology, Johns Hopkins School of Medicine, Baltimore, MD, USA; ^6^ Department of Medicine, Division of Clinical Pharmacology, Johns Hopkins School of Medicine, Baltimore, MD, USA

**Keywords:** prostate cancer, Hedgehog, Sonidegib (LDE-225), *GLI1*, clinical trial

## Abstract

**Purpose:**

To determine the pharmacodynamic effects of Sonidegib (LDE-225) in prostate tumor tissue from men with high-risk localized prostate cancer, by comparing pre-surgical core-biopsy specimens to tumor tissue harvested post-treatment at prostatectomy.

**Methods:**

We conducted a prospective randomized (Sonidegib vs. observation) open-label translational clinical trial in men with high-risk localized prostate cancer undergoing radical prostatectomy. The primary endpoint was the proportion of patients in each arm who achieved at least a two-fold reduction in *GLI1* mRNA expression in post-treatment *versus* pre-treatment tumor tissue. Secondary endpoints included the effect of pre-surgical treatment with Sonidegib on disease progression following radical prostatectomy, and safety.

**Results:**

Fourteen men were equally randomized (7 per arm) to either neoadjuvant Sonidegib or observation for 4 weeks prior to prostatectomy. Six of seven men (86%) in the Sonidegib arm (and none in the control group) achieved a *GLI1* suppression of at least two-fold. In the Sonidegib arm, drug was detectable in plasma and in prostatic tissue; and median intra-patient *GLI1* expression decreased by 63-fold, indicating potent suppression of Hedgehog signaling. Sonidegib was well tolerated, without any Grade 3-4 adverse events observed. Disease-free survival was comparable among the two arms (HR = 1.50, 95% CI 0.26–8.69, *P* = 0.65).

**Conclusions:**

Hedgehog pathway activity (as measured by *GLI1* expression) was detectable at baseline in men with localized high-risk prostate cancer. Sonidegib penetrated into prostatic tissue and induced a >60-fold suppression of the Hedgehog pathway. The oncological benefit of Hedgehog pathway inhibition in prostate cancer remains unclear.

## INTRODUCTION

The Hedgehog (Hh) signaling pathway plays an important role in the embryonic development and homeostasis of many organs and has been implicated in prostate development [1, 2]. The signal transduction pathway is activated when a Hh ligand binds to the trans-membrane Patched (Ptc) receptor, which relieves its inhibitory effect on the Smoothened (Smo) receptor [3]. Activation of the pathway is most reliably indicated by an increase in expression of the Gli transcription factors, such as Gli1, which translocate to the nucleus and control cell proliferation, survival and differentiation [4].

Alterations in Hedgehog pathway components have been linked to the development of human malignancies, particularly basal cell carcinoma, where inhibitors of the Hedgehog pathway, such as the Smo antagonists Sonidegib (LDE-225) and vismodegib, are FDA approved for treatment [5-7]. The regulation and mis-regulation of Hedgehog signaling in prostate cancer is less well known [8, 9]. Upregulation of Hedgehog signaling has been demonstrated in the setting of castration, and in preclinical studies as well as a clinical study utilizing itraconazole (which downregulates Hedgehog signaling) it has been suggested that Hedgehog inhibition may delay the progression of castrate-resistant prostate cancer [10-14]. However, a recently completed human trial of vismodegib failed to show oncological benefit for the treatment of metastatic castration-resistant prostate cancer despite suppression of Hedgehog signaling [15].

The status and importance of Hedgehog signaling in hormone-naïve prostate cancer remains unclear; however enrichment of the Hedgehog pathway as well as other developmental and embryonic stem cell pathways has been reported in clinically localized high grade prostate cancer [9, 16]. Herein we describe a prospective, randomized trial utilizing Sonidegib as a neoadjuvant treatment for high-risk clinically localized prostate cancer. Our primary endpoint was pharmacodynamics, determining whether the Hedgehog pathway is measurable in untreated localized disease and whether Sonidegib treatment penetrates tumor tissue and downregulates Hedgehog signaling as assayed by *GLI1* expression.

## RESULTS

### Patient characteristics

In total, fourteen patients were enrolled in the study from April 2014 through January 2017 (the trial was terminated early by the sponsor). Baseline and post-surgical characteristics of the participants are listed in Table 1. The median age at enrollment was 63 years (range, 50-68). Thirteen of fourteen participants identified as Caucasian/other (92.9%) with one participant identifying as African-American. The median PSA at enrollment was 8 ng/mL (range, 3.1-33.5). The median Gleason sum as determined by core-needle biopsy was 9 (range, 7-10). Only one patient had a Gleason sum < 8 (4+3=7) but his pre-treatment PSA was 22.5 ng/mL, thus satisfying the NCCN high-risk criteria for inclusion [17]. Twelve of fourteen patients had a clinical stage < T3 at the time of enrollment. None of the patients were on systemic therapies prior to enrollment, and all patients had an ECOG performance score of 0.

**Table 1:** Baseline and post-operative characteristics of participants

	Overall (n=14)	Sonidegib (n=7)	No Drug (n=7)
**Age, years**			
Median (range)	63 (50-68)	62 (50-68)	63 (63-66)
**Race (percentage)**			
Caucasian/other	13 (92.9%)	6 (85.7%)	7 (100%)
African-American	1 (7.1%)	1 (14.3%)	0 (0%)
**ECOG Performance status**			
Median (range)	0 (0-0)	0 (0-0)	0 (0-0)
**Pre-treatment PSA, ng/mL**			
Median (range)	8.0 (3.1-33.5)	11.6 (3.1-33.5)	8.0 (3.4-10.8)
**Post-treatment PSA, ng/mL**			
Median (range)	7.9 (2.4-29.7)	12.0 (3.2-29.7)	7.4 (2.4-10.4)
**Biopsy Gleason Sum**			
7	1 (7.1%)	1 (14.3%)	0 (0%)
8	2 (14.3%)	0 (0%)	2 (28.6%)
9	10 (71.4%)	5 (71.4%)	5 (71.4%)
10	1 (7.1%)	1 (14.3%)	0 (0%)
**Clinical Stage**			
T1c	5 (35.7%)	2 (28.6%)	3 (42.9%)
T2a	3 (21.4%)	1 (14.3%)	2 (28.6%)
T2b/T2c	4 (28.6%)	2 (28.6%)	2 (28.6%)
T3	2 (14.3%)	2 (28.6%)	0 (0%)
**Pathologic Gleason Sum**			
7	2 (14.3%)	1 (14.3%)	1 (14.3%)
8	0 (0%)	0 (0%)	0 (0%)
9	11 (78.6%)	5 (71.4%)	6 (85.7%)
10	1 (7.1%)	1 (14.3%)	0 (0%)
**Positive Surgical Margin**	3 (21.4%)	2 (28.6%)	1 (14.3%)
**Extraprostatic Extension**	10 (71.4%)	6 (85.7%)	4 (57.1%)
**Seminal Vesicle Invasion**	5 (35.7%)	3 (42.9%)	2 (28.6%)
**Lymph Node Involvement**	2 (14.3%)	1 (14.3%)	1 (14.3%)
**PSA <0.1 ng/mL at 6 mo**	9 (64.3%)	4 (57.1%)	5 (71.4%)

### Pharmacodynamic analysis

**Figure 1:**
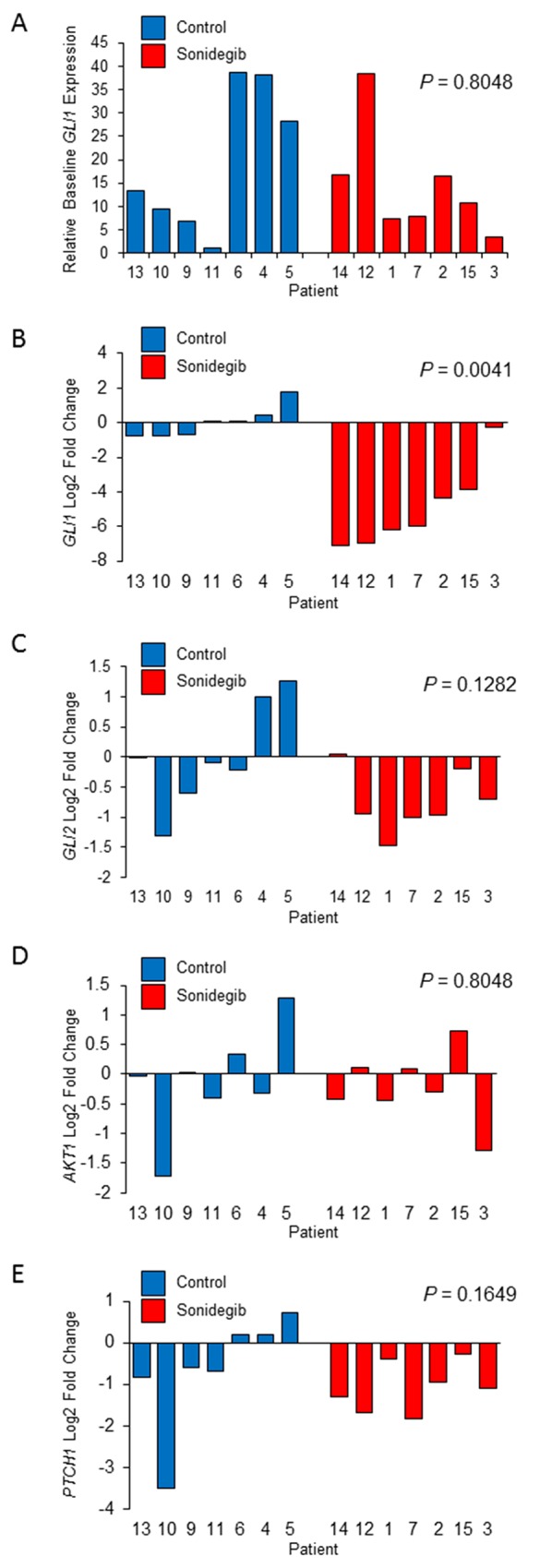
**(A)** Relative levels of baseline GLI1 expression in pre-treatment tumor biopsies as measured by qRT-PCR. B-E) Waterfall plots of Log2 fold change in **(B)** *GLI1*, **(C)** *GLI2*, **(D)** *AKT1*, and **(E)** *PTCH1* mRNA expression in post-treatment tumor tissue compared to baseline as measured by qRT-PCR. Blue color indicates patients in observation group and red color indicates patients in Sonidegib group.

Hh pathway gene expression levels as measured by qRT-PCR in tumor tissue pre- and post-treatment were available for all fourteen patients. The median relative baseline *GLI1* expression level across groups was 11.99 with a high degree of variability between patients (range, 1.00 – 38.53; Figure 1A). There was no significant difference in the relative baseline *GLI1* expression levels between the Sonidegib treatment and observation groups *(P* = 0.80). The median baseline *GLI1* C_t_ value across groups was 31.18 (range, 28.34-34.31) compared to a median baseline C_t_ value of 30.78 for the housekeeper gene, *HPRT1* (range, 29.03-33.51). *GLI1* expression was significantly reduced in the Sonidegib treatment group (median decrease of 63-fold, range 135-fold decrease to 1.2-fold decrease) vs. the observation group (median increase of 1.0-fold, range 1.7-fold decrease to 3.4-fold increase) (*P* < 0.01; Figure 1B) with 6 of 7 patients (86%) in the Sonidegib group achieving a >2-fold *GLI1* reduction, while this did not occur in any of the 7 patients in the control group.

*GLI2* expression was also generally reduced in the Sonidegib group (median decrease of 1.9-fold, range 2.8-fold decrease to 1.0-fold increase) vs. the observation group (median decrease of 1.1-fold, range 2.5-fold decrease to 2.4-fold increase), and this difference approached statistical significance (P = 0.13) (Figure 1C). There was not a significant difference between the two groups in fold change of *AKT1* expression, with a median decrease of 1.2-fold in the Sonidegib treatment group (range, 2.5-fold decrease to 1.7-fold increase) vs. a median decrease of 1.0-fold in the observation group (range, 3.3-fold decrease to 2.5-fold increase) (P = 0.80) (Figure 1D). In addition, there was not a significant difference between the two groups in fold change of *PTCH1* expression, with a median decrease of 2.1-fold in the Sonidegib treatment group (range, 3.6-fold decrease to 1.2-fold decrease) vs. a median decrease of 1.5-fold in the observation group (range, 11.4-fold decrease to 1.6-fold increase) (P = 0.16) (Figure 1E).

### Pharmacokinetic analysis

**Figure 2:**
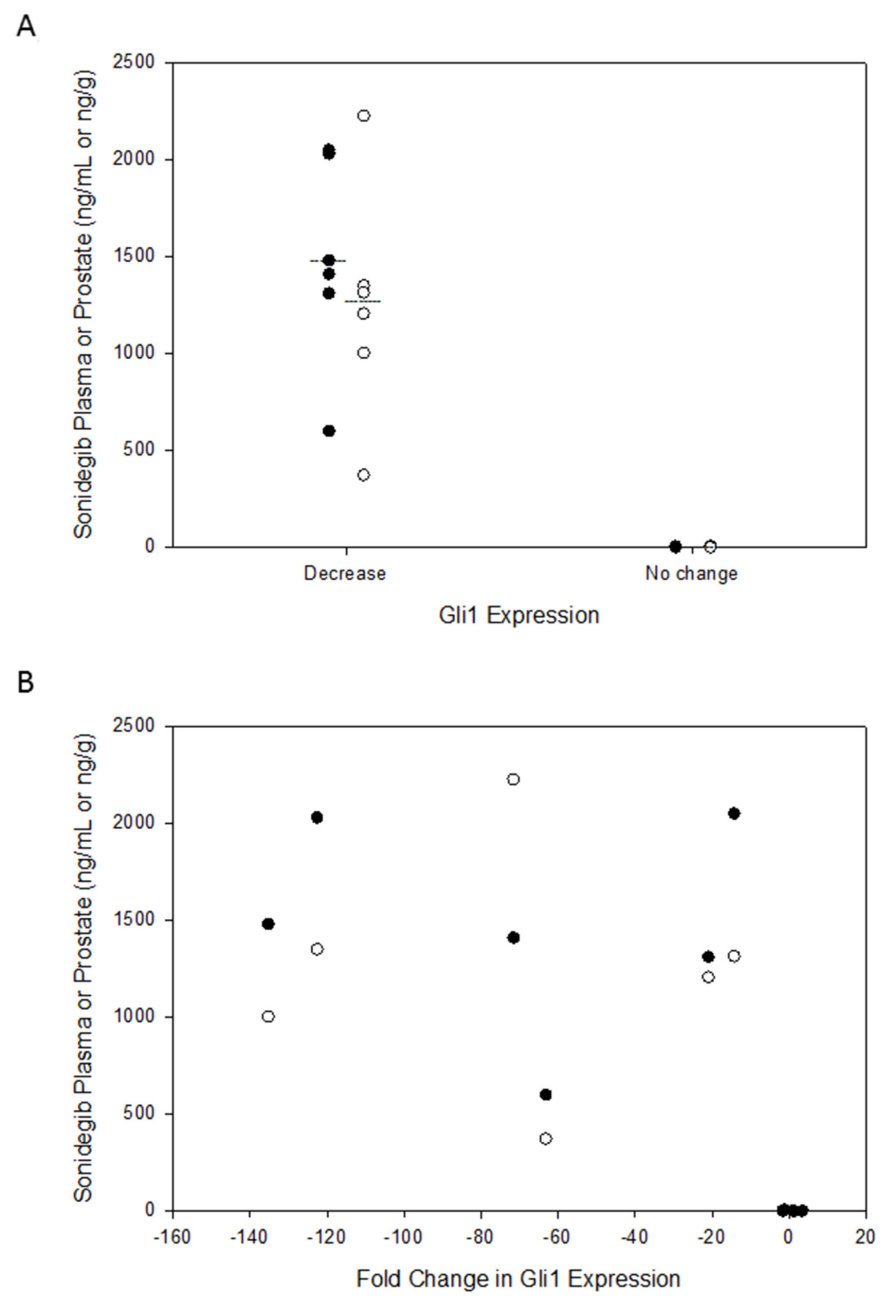
**(A)** Relationship between GLI1 expression changes and individual Sonidegib concentrations in prostatectomy tumor tissue (open circles, expressed as ng/g) and peripheral plasma (closed circles, expressed as ng/mL). Mean concentrations for each matrix is noted by the solid line. **(B)** Fold change in *GLI1* expression and individual Sonidegib concentrations in prostatectomy tumor tissue (open circles, expressed as ng/g) and peripheral plasma (closed circles, expressed as ng/mL).

Sonidegib was detectable in plasma and prostate tumor tissue in all seven patients who received 28 days of continuous daily dosing of 800 mg. One of the seven patients (#3) who received 800 mg had extremely low drug concentrations in plasma (4 ng/mL) and prostate tumor tissue (6 ng/g). This patient also did not have a marked decrease in *GLI1* expression at prostatectomy (Figure 1B, patient #3). In the remaining six patients, the average Sonidegib exposure in plasma and prostate tumor tissue was 1480±536 ng/mL and 1245±600 ng/g, respectively. Sonidegib was not detected in any of the seven patients in the control group. There was a significant correlation between Sonidegib exposure and a decrease in *GLI1* expression in both plasma (*P* =0.0009) and prostate tumor tissue (*P*=0.0009) (Figure 2).

### Clinical outcomes

**Figure 3:**
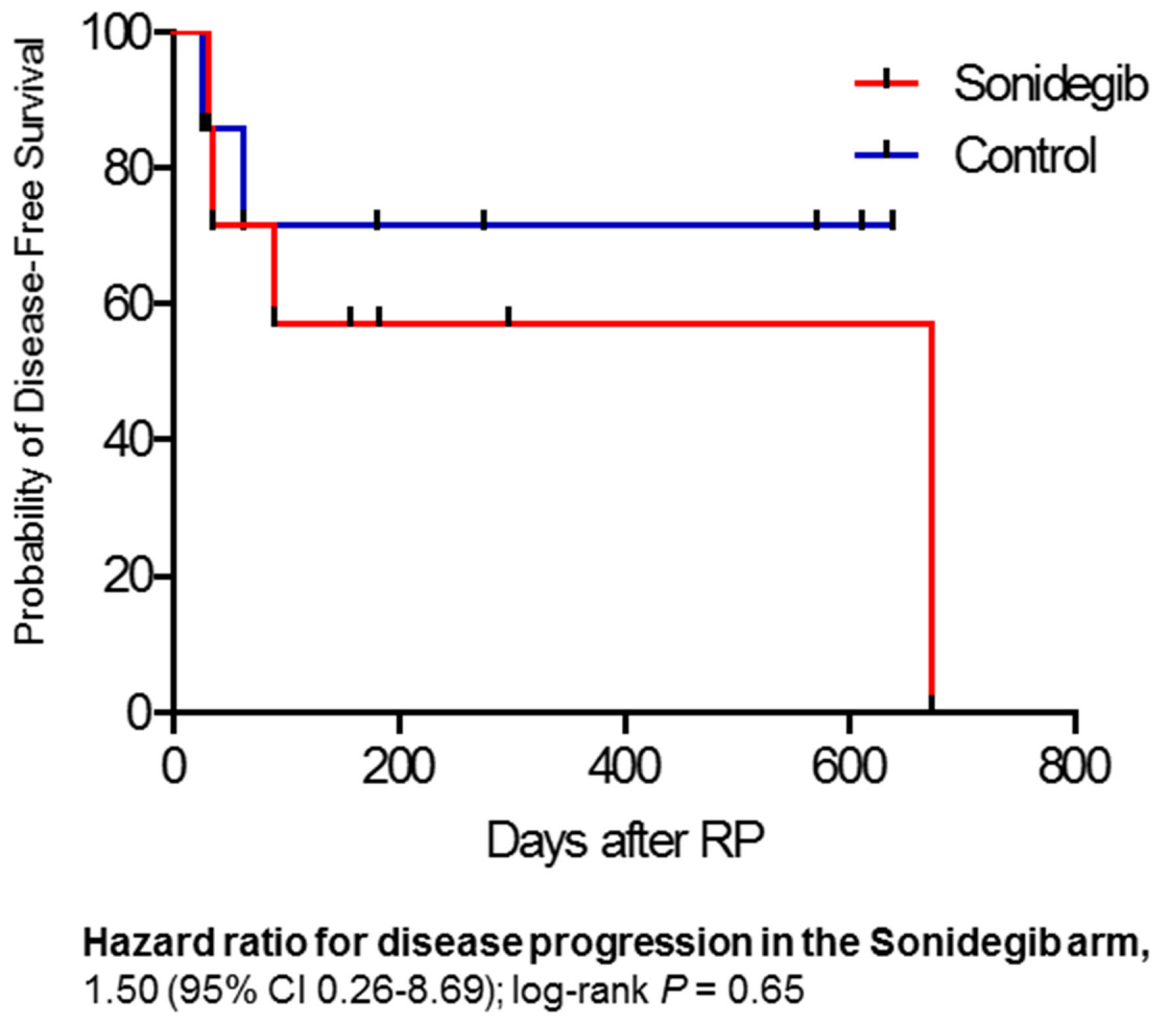
Kaplan-Meier curve of disease-free survival Observation group indicated in blue color and Sonidegib treatment group indicated in red color.

All fourteen patients were evaluable for PSA response before undergoing prostatectomy. After 4 weeks of treatment (Sonidegib vs. observation groups), neither group experienced a significant change in serum PSA levels. Post-treatment, the Sonidegib group had a median increase in PSA of 0.4 ng/mL (range, -16.8 to 7.2) while the observation group had a median decrease in PSA of 1 ng/mL (range, -1.7 to 0.9). At the time of radical prostatectomy, two of seven Sonidegib participants (29%) and one of seven observation-group participants (14%) had positive surgical margins, six of seven Sonidegib (86%) and four of seven observation (57%) group participants had extraprostatic extension, three of seven Sonidegib (43%) and two of seven observation (29%) group participants had seminal vesicle invasion, and one patient from each group (14%) had positive lymph nodes (Table 1). The median overall pathologic Gleason sum at the time of radical prostatectomy was 9 (range, 7-10) with participants in the Sonidegib treatment group having a median sum of 9 (range, 7-10) and participants in the observation group having a median sum of 9 (range, 7-9). At three and six months post-surgery, four of seven Sonidegib treatment group participants (57%) and five of seven observation group participants (71%) had PSA measurements remaining < 0.1 ng/mL. At the time of data cutoff, with a median follow-up of 181.5 days, disease progression (PSA ≥ 0.2 ng/mL) had occurred in four of seven Sonidegib participants (57%) and two of seven observation-group participants (29%) (Figure 3). There was no significant difference in disease-free survival between Sonidegib treatment group participants and observation group participants (Hazard ratio 1.50, 95% CI 0.26–8.69, *P* = 0.65).

### Safety

Sonidegib was well tolerated without any observed grade 3 or 4 events. The most common adverse events included nausea, increased CK levels, AST/ALT elevation, fatigue, dry mouth, arthralgia, dysgeusia, myalgia, and musculoskeletal pain, which is consistent with the known toxicity profile of Sonidegib [18]. There were no new safety concerns noted in this study. Several common toxicities noted in other trials of Sonidegib, including alopecia, anorexia, and weight loss, did not occur in this study. A complete summary of all adverse events is listed in Table 2. All patients in both study arms met the routine perioperative and postoperative parameters as determined by their urologic surgeon. Treatment with Sonidegib did not impact perioperative/postoperative events such as complications, readmissions, length of stay, time to drain removal, or time to catheter removal.

**Table 2:** Adverse events

Adverse event	Overall (n=14) Grades 1-2		Sonidegib (n=7) Grades 1-2		No Drug (n=7) Grades 1-2	
	Number	%	Number	%	Number	%
**Nausea**	3	21%	3	43%	0	0%
**ALT elevation**	2	14%	2	29%	0	0%
**CK increase**	2	14%	2	29%	0	0%
**Fatigue**	2	14%	1	14%	1	14%
**Arthralgia**	1	7%	1	14%	0	0%
**AST elevation**	1	7%	1	14%	0	0%
**Dry Mouth**	1	7%	1	14%	0	0%
**Dysgeusia**	1	7%	1	14%	0	0%
**Myalgia**	1	7%	1	14%	0	0%
**Pain, musculoskeletal**	1	7%	1	14%	0	0%
**Pain, other**	1	7%	1	14%	0	0%
**Hyperglycemia**	1	7%	0	0%	1	14%
**Thrombocytopenia**	1	7%	0	0%	1	14%

## DISCUSSION

Herein we describe a pharmacodynamic neoadjuvant study of the small-molecule Smo antagonist, Sonidegib (LDE-225), in clinically localized, treatment naïve, high- and very-high risk prostate cancer. To our knowledge, this represents the first study of a targeted small molecule Hedgehog pathway inhibitor in clinically localized prostate cancer.

This trial demonstrated several important findings. First, we identified detectable levels of *GLI1* in the majority of patients with clinically localized high-risk prostate cancer and more than half of the patients had *GLI1* threshold cycle (C_t_) values comparable to the C_t_ values of a housekeeping gene, *HPRT1*. This suggests active Hedgehog signaling in at least a subset of hormone-naïve high-risk disease. Secondly, we observed consistent and meaningful downregulation of *GLI1* expression upon exposure to targeted Smo antagonism. Over 10-fold downregulation was seen in six of the seven patients randomized to neoadjuvant Sonidegib therapy, and in all of the patients in whom appreciable tissue and blood levels of Sonidegib were identified. *GLI2* expression levels also generally decreased following neoadjuvant Sonidegib therapy but to a lesser degree. Finally, we found that Sonidegib was well tolerated and did not result in significant toxicity or interfere with peri-surgical or post-surgical recovery.

Our trial was not designed to determine whether Sonidegib could exert direct anti-tumor effects or whether a short course of neoadjuvant treatment could alter oncological outcomes or recurrence rates. Regardless, we saw no striking treatment effect when examining radical prostatectomy specimens among Sonidegib-treated and control groups, and witnessed no significant difference in disease-free survival among the two study groups. A recent phase II trial in castrate-resistant metastatic prostate cancer utilizing vismodegib also failed to show oncological benefit for treatment of advanced prostate cancer with Smo antagonists [15]. These two studies suggest that, at least as a monotherapy, Hedgehog pathway inhibition might have limited utility in localized or metastatic prostate cancer. Further exploratory analyses will be needed to identify pathways that are associated with Hedgehog suppression and whose inhibition might allow for rational therapeutic synergy with Smo antagonists in the treatment of prostate cancer.

This study has some limitations, but also several strengths. The most notable shortcoming was our inability to complete enrollment as initially planned, due to premature closure of the study by the sponsor. This would have clearly decreased our ability to interpret the primary endpoint if the degree of *GLI1* mRNA suppression in the treatment arm was modest compared to the control arm. However, due to the striking *GLI1* suppression that we observed with Sonidegib (median >60-fold reduction from baseline) and the relative absence of *GLI1* modulation in the control arm, we believe that the study adequately demonstrated an unequivocal pharmacodynamic effect of the study drug as initially hypothesized. While drug administration was facilitated by the oral bio-availability of Sonidegib, another limitation was that patient compliance with therapy could not be strictly enforced. For example, it is not clear whether the one patient randomized to study drug who lacked drug in their blood and prostate and was the only patient randomized to Sonidegib without down-regulation of *GLI1* expression simply was not compliant with the neoadjuvant drug regimen. Another limitation was the inability to draw any clinical conclusions about the anti-tumor activity of Sonidegib or its effect on disease recurrence rates following prostatectomy. However, a major strength of this study was our ability to assess intra-patient changes in *GLI1* levels by interrogating pre-treatment and post-treatment tumor tissue in each individual patient. This approach is clearly preferable to simply comparing the mean or median *GLI1* expression in post-treatment tumor samples in Sonidegib-treated patients *versus* controls, which would not account for dynamic changes in the biomarkers of interest induced by Sonidegib treatment.

Discrepancies between preclinical data and clinical observations signify the need to carry out pharmacodynamic studies of promising cellular pathway inhibitors before conducting large-scale efficacy studies. From a tissue acquisition perspective, the neoadjuvant setting can be ideal for the study of novel therapies. In this study, we show that administration of the small molecule Smo antagonist Sonidegib is well tolerated and effectively penetrates prostate tissue, where it downregulates key nodes of the Hedgehog pathway. In this relatively small study, we did not see obvious anti-neoplastic effects of Hedgehog inhibition on prostate cancer. Further exploratory analysis of the tissue from this trial (i.e. comparisons of genome-wide mRNA expression data between groups) may help determine key components of the Hedgehog pathway in clinically localized prostate cancer and suggest opportunities for combination of Smo antagonists with other systemic therapies.

## MATERIALS AND METHODS

### Patients

This study (NCT02111187) enrolled men with NCCN high- or very-high risk localized prostate cancer prior to radical prostatectomy [17]. A diagnosis of prostate cancer was confirmed if histologically-documented prostatic adenocarcinoma was found in ≥ 2 biopsy cores. High-risk patients were identified as having at least one of the following NCCN high-risk features at the time of initial screening: Gleason sum ≥8 as determined by core-needle biopsy, serum PSA levels >20 ng/mL, clinical stage ≥T3 [17]. All patients were required to be at least 18 years of age. All patients had an ECOG score ≤ 2 at the time of enrollment and no evidence of known metastatic disease (M0 or Mx allowed). Other eligibility criteria included adequate bone marrow (ANC ≥1500/μL, platelet count ≥100,000/μL, Hgb ≥9g/dL), liver (serum total bilirubin ≤1.5 x the upper limit of normal [ULN], AST and ALT ≤2.5 x ULN), and renal function (serum creatinine ≤1.5 x ULN or 24-hour creatinine clearance ≥50mL/min, plasma creatine kinase [CK] <1.5 x ULN if known). Patients were counseled not to embark on any new strenuous exercise regimen after initiation of Sonidegib treatment to prevent significant increases in plasma CK levels.

Study exclusion criteria included major surgery within 4 weeks of enrollment, inability to swallow oral medications, concurrent treatment with anti-neoplastic agents (e.g. chemotherapy, targeted therapy, radiotherapy) or any other investigational agents, or prior therapy with Sonidegib or other Hh pathway inhibitors. Study exclusion criteria also included patients with neuromuscular or muscular disorders (e.g. inflammatory myopathies, muscular dystrophy, ALS, spinal muscular atrophy) or impaired cardiac function or significant heart disease (e.g. angina pectoris or acute myocardial infarction within 3 months of study enrollment, QTc > 450msec on the screening ECG, past medical history of clinically significant ECG abnormalities, family history of prolonged QT-interval syndrome, heart failure, uncontrolled/labile hypertension). While on study, patients could not be treated concomitantly with anticoagulants (e.g. warfarin), moderate/strong inhibitors or inducers of CYP3A4/5, drugs metabolized by CYP2B6 or CYP2C9 that have a narrow therapeutic index, or drugs known to cause rhabdomyolysis (e.g. statins [except pravastatin, which was permitted], fibrates). All patients were required to provide written informed consent prior to any screening procedures.

### Study design

**Figure 4:**
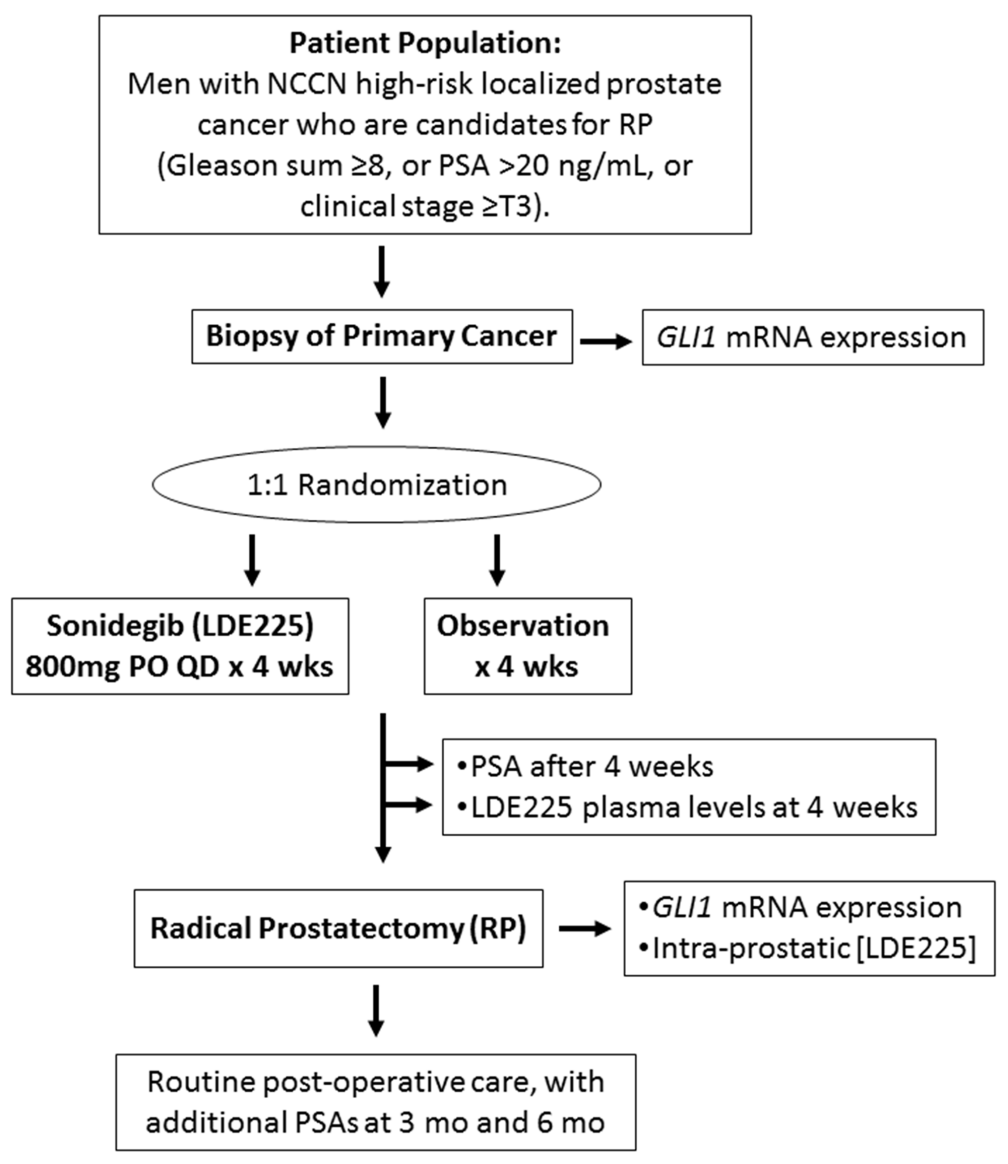
Study schema

The study schema is outlined in Figure 4. This was a single-institution randomized two-arm (Sonidegib vs. observation) open-label prospective clinical trial in men with high-risk localized prostate cancer undergoing radical prostatectomy. At the time of study enrollment, all patients underwent a research tumor biopsy. Men were then randomly assigned (1:1) to receive oral Sonidegib (800mg daily) or observation for four weeks (± 3 days) prior to prostatectomy. The last dose of Sonidegib was taken on the morning prior to prostatectomy with the surgery being performed within ∼24 hours of the last dose. After four weeks, patients had a post-treatment PSA measurement and blood sample collection to determine plasma Sonidegib levels. All patients then underwent radical prostatectomy (with bilateral pelvic lymphadenectomy as appropriate), at which point two 250mg biopsies of prostate tissue were obtained, frozen, and stored for analysis. All patient samples were stored at -70°C or below until analysis. Patients underwent routine post-operative care, with repeat PSA testing at 3 and 6 months post-surgery for disease evaluation. The study was approved by the Johns Hopkins Institutional Review Board and was registered on www.clinicaltrials.gov (NCT02111187).

### Study outcomes

The primary study outcome was a change from baseline in tissue *GLI1* mRNA expression levels using quantitative real-time PCR (qRT-PCR) analysis in each group (Sonidegib and observation). This was defined as the proportion of patients who achieved at least a two-fold reduction in *GLI1* expression in post-treatment *vs.* pre-treatment tumor tissues. In addition, changes in other markers in the Hh pathway (*GLI2*, *PTCH1*, *AKT1*) were also investigated. Plasma and tissue drug levels were also assessed. Secondary outcomes included whether pre-surgical treatment with Sonidegib diminished the risk of disease progression (PSA ≥ 0.2 ng/mL) following radical prostatectomy, and the number of patients with adverse events in each group (Sonidegib and observation). Safety and tolerability, including any drug-related toxicities of Sonidegib, were reported via CTCAE version 4.0.

### Pharmacodynamic analysis

Pre- and post-treatment tumor biopsies were evaluated by qRT-PCR for changes to Hh-regulated transcripts, including *GLI1*, *GLI2*, *AKT1* and *PTCH1*. RNA from fresh-frozen tissue was isolated using the Qiagen RNeasy Micro Kit (Cat#774004). First-strand complementary DNA was synthesized from RNA with RT2 You Prime First Strand Beads (GE Healthcare, Cat#27926401) with random hexamer primers (Invitrogen, Cat#N808127). Quantitative RT-PCR was performed in triplicate for each specimen on a StepOnePlus Real-Time PCR System (Applied Biosystems, Cat#4376600) using Taqman reactions (Taqman Universal Master Mix II, Life Technologies, Cat#4440040) with primers for *GLI1* (Hs02800695_ m1), *GLI2* (Hs01119974_m1), *AKT1* (Hs00178289_m1), or *PTCH1* (Hs00181117_m1), and normalized to *HPRT1* (Hs02800695_m1). Applied Biosystems software was used to calculate threshold cycle (C_t_) values for each gene of interest.

### Pharmacokinetic analysis in plasma and tissue

Sonidegib concentrations in plasma were determined by a validated high-performance liquid chromatography with mass spectrometry (LC/MS/MS) detection method over the range of 0.025-250 ng/mL with a 1:10 dilution allowing for quantitation to 2500 ng/mL. For tissue-based analyses, fresh frozen tissue samples were homogenized in plasma to yield a tissue homogenate concentration of 200 mg/mL. The homogenate was further diluted in plasma (1:1, v/v). Sonidegib concentrations in tissue homogenate were determined by a validated LC/MS/MS method over the range of 0.025-250 ng/mL. Prostate tumor tissue samples were then quantitated in ng/g as: nominal concentration (ng/mL) x 6 (standardized dilution of 200 mg/mL) x any additional dilution factor (up to 1:10).

### Statistics and sample size

The primary endpoint was to assess a meaningful change in tissue *GLI1* mRNA expression levels using qRT-PCR analysis in each group (Sonidegib and observation), defined as at least a 2-fold intra-patient reduction in *GLI1* expression in post-treatment *vs.* pre-treatment tumor tissues. We hypothesized that this degree of *GLI1* suppression would be achieved in <10% of men in the control arm (null hypothesis) and in ≥70% of men in the Sonidegib arm (alternative hypothesis). Using a 2-sided alpha (α) of 0.10 and beta (β) of 0.20 (power = 80%), a sample size of 11 patients/arm (total = 22 men) would be required to observe a difference in the primary endpoint from <10% (control arm) to ≥70% (Sonidegib arm). The trial would be considered a success if ≥70% of men in the Sonidegib arm achieved this primary endpoint.

Baseline participant characteristics, clinical outcomes, and drug concentrations in plasma and tumor tissue were reported using descriptive statistics. Fold changes from baseline in *GLI1*, *GLI2*, *AKT1*, and *PTCH1* tissue expression were reported using descriptive statistics and the Wilcoxon rank-sum test. A Mann–Whitney U-test was used to assess correlations between Sonidegib exposure in plasma or prostate tumor tissue and alterations in *GLI1* expression. The *a priori* level of significance was set at P < 0.05. Statistical analyses were performed using GraphPad Prism (version 5.00 for Windows, GraphPad Software, La Jolla, California) and JMP™ statistical discovery software (SAS Institute, Cary, North Carolina). The Probability of Disease-Free Survival is defined as the time from radical prostatectomy until disease progression (PSA ≥ 0.2 ng/mL), and results were summarized using the Kaplan-Meier method and the log-rank test.

## Abbreviations

ALT: alanine transaminase
ANC: absolute neutrophil count
AST: aspartate transaminase
CK: creatine kinase
ECG: electrocardiogram
ECOG PS: Eastern Cooperative Oncology Group Performance Score
Hgb: Hemoglobin
Hh: Hedgehog
NCCN: National Comprehensive Cancer Network
PSA: prostate-specific antigen
Ptc: Patched
qRT-PCR: quantitative real-time polymerase chain reaction
Smo: Smoothened
ULN: upper limit of normal
